# Factors influencing the implementation of integrated management of childhood illness (IMCI) by healthcare workers at public health centers & dispensaries in Mwanza, Tanzania

**DOI:** 10.1186/1471-2458-14-277

**Published:** 2014-03-25

**Authors:** Augustine Kiplagat, Richard Musto, Damas Mwizamholya, Domenica Morona

**Affiliations:** 1 Zonal Health Resource Centre-Lake Zone, Mwanza, Tanzania; 2 Department of Community Health Sciences, University of Calgary, Calgary, AB, Canada; 3 Department of Child Health & Paediatrics, Catholic University of Health and Allied Sciences-Bugando, Mwanza, Tanzania; 4 Department of Parasitology & Entomology, Catholic University of Health and Allied Sciences-Bugando, Mwanza, Tanzania

**Keywords:** Lower level health facilities, Integrated Management of Childhood Illness (IMCI), Council Health Management Team (CHMT), Factors influencing the implementation of IMCI

## Abstract

**Background:**

Integrated Management of Childhood Illness (IMCI) was developed by the World Health Organization (WHO) and the United Nations International Children’s Fund (UNICEF) and aims at reducing childhood morbidity and mortality in resource-limited settings including Tanzania. It was introduced in 1996 and has been scaled up in all districts in the country. The purpose of this study was to identify factors influencing the implementation of IMCI in the health facilities in Mwanza, Tanzania since reports indicates that the guidelines are not full adhered to by the healthcare workers.

**Methods:**

A cross-sectional study design was used and a sample size of 95 healthcare workers drawn from health centers and dispensaries within Mwanza city were interviewed using self-administered questionnaires. Structured interview was also used to get views from the city IMCI focal person and the 2 facilitators. Data were analyzed using SPSS and presented using figures and tables.

**Results:**

Only 51% of healthcare workers interviewed had been trained. 69% of trained Healthcare workers expressed understanding of the IMCI approach. Most of the respondents (77%) had a positive attitude that IMCI approach was a better approach in managing common childhood illnesses especially with the reality of resource constraint in the health facilities. The main challenges identified in the implementation of IMCI are low initial training coverage among health care workers, lack of essential drugs and supplies, lack of onsite mentoring and lack of refresher courses and regular supportive supervision. Supporting the healthcare workers through training, onsite mentoring, supportive supervision and strengthening the healthcare system through increasing access to essential medicines, vaccines, strengthening supply chain management, increasing healthcare financing, improving leadership & management were the major interventions that could assist in IMCI implementation.

**Conclusions:**

The healthcare workers can implement better IMCI through the collaboration of supervisors, IMCI focal person, Council Health Management Teams (CHMT) and other stakeholders interested in child health. However, significant barriers impede a sustainable IMCI implementation. Recommendations have been made related to supportive supervision and HealthCare system strengthening among others.

## Background

The Integrated Management of Childhood Illness (IMCI) is a strategy which was developed by the World Health Organization (WHO) and the United Nations International Children’s Fund (UNICEF) in 1992 as an integrated approach to improve child health [1]. IMCI is a set of integrated (combined) guidelines, instead of separate guidelines for each illness which can affect a child. Its main objective is the reduction of mortality and morbidity associated with the major causes of childhood illness. According to UNICEF, in the year 2010 about 7.6 million children died before reaching their fifth birthday. Most child deaths (and 70% in developing countries) result from one the following five causes or a combination thereof: acute respiratory infections, diarrhea, measles, malaria and malnutrition [1,2].

The IMCI strategy has been adopted in over 100 countries, including Tanzania. A study in Tanzania indicates that, although all the districts have received training on IMCI, the main challenge to implementation is poor adherence to the guidelines with most of the health care workers trained in IMCI not following the protocol consistently [3] Another study in Tanzania found that health care workers diagnose children in terms of a single disease and prescribe accordingly. Referral practices are also poor, with less than 50% of severely ill children being referred [4].

The IMCI initiative is in line with the Millennium Development Goal (MDG) number four which aims at reducing under 5 mortality rate by two thirds by 2015. If challenges and factors influencing the implementation of IMCI case management guidelines among health care workers are addressed then it will contribute to the achievement of MDG number four. Since its introduction, IMCI has achieved impressive results both in reducing childhood mortality and in improving the quality of life of children in Tanzania [5]. Under-five mortality decreased from 191 per thousand live births in 1990 to 133 in 2005 and further to 81 in 2010 in the Tanzania Mainland [6]. This is a decrease of 58% but still short of the 66% target. Nevertheless a number of challenges related to IMCI implementation remain, in particular non-adherence to IMCI guidelines among health care workers. Addressing those challenges could further reduce the child mortality and lead to achievement of MDG 4.

**Figure 1:**
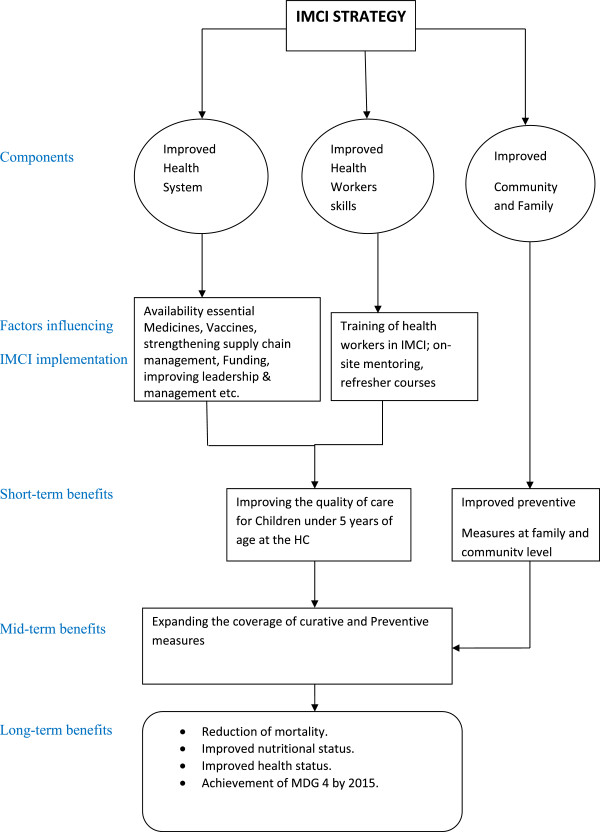
Conceptual framework.

According to WHO [1,2,7-11] the IMCI improves health worker performance on the management of childhood illness therefore reducing mortality and morbidity. However, a study in Tanzania [3,12] has shown that many health care workers do not adhere to IMCI guidelines and the reasons for this remain unclear. Decision-making by health care workers may be shaped by economic, patient-related, training, professional and organizational factors as summarized in conceptual framework in Figure 1. Understanding non-adherence will help policy and decision makers to improve IMCI implementation.

This study was therefore carried out to identify the factors influencing the implementation of IMCI at Public health centers & dispensaries in Mwanza city.

## Methods

The study was conducted in health facilities in Mwanza city located in Northwest of Tanzania, southern Lake Side of Lake Victoria in East Africa. Mwanza region is among the regions in Tanzania with high under-five mortality rates [13-15] and over 75% of the under 5 population are served in these government owned health facilities, therefore this was the compelling reason why the government owned facilities were chosen for this study. A cross-sectional study design was used and the study population was made up of all health care workers working in government owned health centers & dispensaries in Mwanza city.

As per Mwanza city health department statistics (January 2010), there are 28 operational government owned health centers & dispensaries offering curative services to children among other services. There are 23 dispensaries and 5 health centers with a total of 248 health care workers. Out of this 138 healthcare workers are in Nyamagana district while 110 healthcare workers are working in Ilemela district. As per the Ministry of Health Establishment, Health centers and dispensaries are manned by enrolled nurses, clinical assistants, clinical officers, registered nurses and assistant medical officers while other cadres including medical officer and graduate nurses were not employed in these health facilities because this category is usually employed at District Hospital or above. A total of 95 health care workers drawn from the 28 health facilities (dispensaries & health centers) in Mwanza city council were selected for interview. The 95 healthcare workers were arrived at as the sample size based on the formula calculation of a sample size where prevalence is not required or known [16].

The formula is as follows:

$$n=N/1+N\;\left(e\right)2$$

Where:

n = Sample size.

N = Population size

e = Sampling error (which is assumed to be 5%) i.e. 95% Confidence interval.

n = 245/1 + 245(0.05)2 = 152.

However a sample size of 95 was taken. (This represents 40% of the study population). The major reason for scaling down the sample size was due to resource constrain, therefore making the study to be underpowered. A larger sample might have revealed more significant relationships. However, the sample size of 95 is large for a mixed methods study with resource constraints.

Then a Multistage sampling process was used in 2 stages:

**Stage 1:** Simple random sampling; at this stage simple random sampling was used to select 10 government owned dispensaries out of the 23 in Mwanza city. This was done by writing all dispensaries on pieces of papers, then randomly picking up 10 which become the sample. All the 5 health centers were selected. This led to having 15 health facilities (Health Centers & dispensaries) for the study.

**Stage 2:** Convenience sampling; the healthcare workers present at the selected health facilities during the day of interview were selected to participate in the study. Enrolled Nurses and Clinical Officers were highly represented since they were the majorly present in most of the Health Facilities visited. They were selected based on their availability at work place and willingness to participate in the study.

The methods of data collection were self-administered questionnaires for health care workers; the questionnaires were administered then collected after 2 days from the day of administration. An in-depth interview for the Mwanza city IMCI focal person, selected IMCI 2 facilitators and health facilities administration were also used. Both English and Swahili versions of the questionnaires were used. The Types of data collected were both Quantitative data and Qualitative data.

The questionnaires were pretested at Missungwi health Centre before data collection of the study. All ambiguous questions were deleted and unclear ones rephrased. A research assistant was trained on all the data collection tools and entry. Questionnaires were translated from English to Swahili by one colleague and then from Swahili to English by another different colleague to check the consistency and minimize change of meaning of the questions.

Quantitative data included the number of healthcare workers working at the health facilities, years of experience, level of education, number of IMCI follow-up among others. The method of data collection used in this category was self-administered questionnaires. Quantitative data collected were double entered into and analyzed using SPSS software version 17. Descriptive statistics were summarized in percentages. Categorical data were tested for significance using Chi-square. A P-Value less than 0.05 was considered statistically significance.

Qualitative data included an in-depth interview with key informants from the healthcare facilities administration, Mwanza city IMCI focal person, and direct observations. The in-depth interview guide with open-ended questions was used to probe the participants on IMCI implementation issues. The qualitative responses from key informants were written in notebooks. There was no audio-recording due to its intrusiveness and this could discourage active participation from the key informants. Qualitative data were analyzed according to the content themes. Data was entered into password protected database to maintain confidentiality throughout the entire process. The findings obtained were presented using tables and graphs. The findings are discussed in depth and all possible explanations and inferences reported.

### Ethical considerations

In the study the following ethical issues were adhered to:

1. The Ethical clearance to conduct the research was obtained from the joint CUHAS/BMC Research and Publications Committee.

2. The consent to proceed with the research was obtained from the City Council Medical Officer of Health & selected health facilities administration.

3. The purpose of the study was explained to the respondents and written consent obtained before proceeding with the study.

4. The information obtained was kept private, confidential and anonymous.

5. No names were asked for; Serial numbers in the questionnaires were used for analysis purposes only.

## Results

This section presents the quantitative and qualitative findings from the healthcare workers related to the factors associated with the implementation of IMCI.

From Table 1, 44 (46%) of the health care workers were working in health centers while 51 (54%) were from dispensary level. The highest percentage of health care workers interviewed were enrolled nurses 42 (44%), followed by clinical officers 26 (27%), registered nurses 14 (15%), assistant medical officers 7 (8%) and clinical assistants which represented 6 (6%) of the sample. The other cadres including medical officer and graduate nurses were not employed in these health facilities because this category is usually employed at Hospital level as per the Ministry of Health establishment. Majority of the healthcare workers had clinical experience of over 10 years (62%), followed by those of 2–5 years’ experience (27%), and then 6–10 years’ experience (6%) while the health workers with less than 1 year experience were only 4%.

**Table 1:** Distribution of participating health care workers by facility type

**Health facility**	**Frequency**	**Percent**
Health Centre	44	46
Dispensary	51	54
Total	95	100

Sixty-nine percent of the health care workers who had received IMCI training felt that the training was not adequate and they suggested that on site mentoring and refresher courses were needed to complement the training received from a workshop setting. The remaining 31% of trained health care workers felt that the training they received was adequate to implement IMCI successfully. All of the health care workers trained reported that they had never received IMCI refresher courses since their original IMCI training. The health care workers who had been trained expressed interest in attending IMCI refresher courses and updates especially on malaria case management and care and treatment of children living with HIV & AIDS.

**Figure 2:**
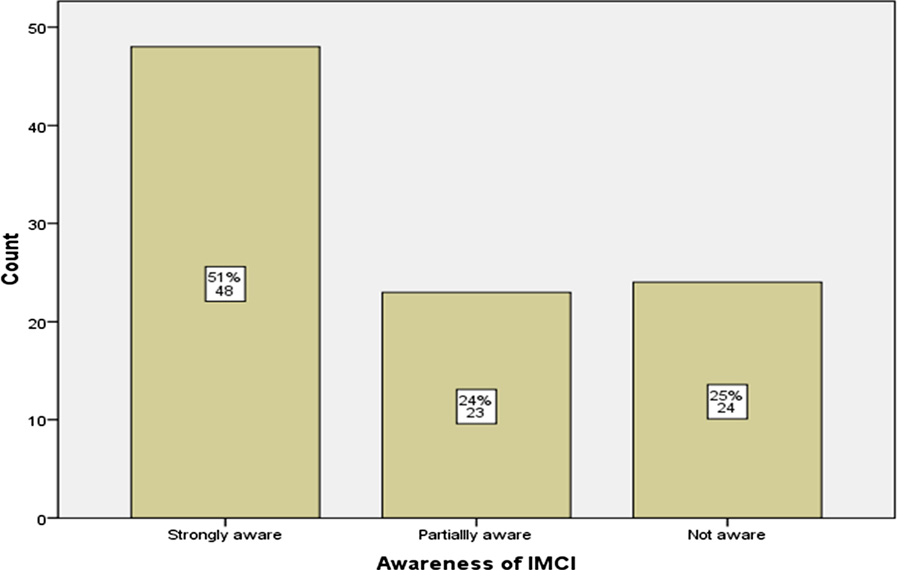
Awareness of IMCI.

About half of the health care workers (51%) interviewed were knowledgeable of the IMCI approach and the common childhood illness where IMCI guidelines need to be followed. The childhood illness mentioned by health workers, in which the IMCI guidelines are followed, included malaria, diarrhea, measles, respiratory conditions, and malnutrition. Figure 2 below shows awareness the IMCI of Healthcare workers interviewed.

### Year attended the training

**Figure 3:**
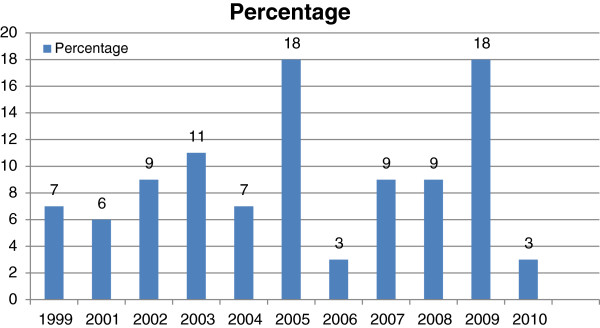
Year of training.

The health care workers who had attended IMCI training were trained between 1999 and 2011 as shown in Figure 3 below.

### Comparison of the length of the training and IMCI awareness

As summarized in Table 2, majority of the health care workers who attended training (73%) went for 2 weeks training, which was significantly associated with an increased awareness about IMCI, followed by 13% who attended 1 week IMCI training, 6% who attended less than 3 days training, 4% who attended the IMCI training in 3–5 days while the remaining 4% received the training of more than 2 weeks duration. The relationship between length of the IMCI training and awareness of IMCI among the health care workers was statistically significant (chi-square with ten degrees of freedom = 95.000, p = 0.000).

**Table 2:** Awareness of IMCI and the length of the training cross tabulation

		**The length of the training**						**Total**
		**Not trained**	**Less than 3 days**	**3-5 days**	**1 week**	**2 weeks**	**More than 2 weeks**	
Awareness of IMCI	Not aware	24	0	0	0	0	0	24
	Partially aware	23	0	0	0	0	0	23
	Strongly aware	0	3	2	6	35	2	48
Total		47	3	2	6	35	2	95

Out of 48 health care workers trained on IMCI, follow up was done for only 20 (42%). The remaining 58% were never followed up after the training. Most of the health care workers who received follow up were done within 1–6 months after the training. Only 40% of the healthcare workers had an opportunity to be followed at least twice. 55% of the health care workers interviewed reported that they follow the IMCI guidelines in their work place since it is a requirement to do so. The MOHSW advocates for treatment of childhood illness by following the IMCI guidelines but not all healthcare workers follow them consistently.

The challenges expressed by healthcare workers in the implementation of IMCI in their facilities includes the following; according to the new guidelines in the treatment of Malaria where the child with fever is not allowed to be given ACT (Artemisin Combined Therapy) until Malaria Rapid Diagnostic test (MRDT) test is done and results is positive (reactive) and therefore the IMCI need to be updated to reflect these changes. Also IMCI still recommend SP but the latest new guidelines on malaria recommend ACT. Shortage of essential drugs especially for treating pneumonia, diarrhea (ORS) and malaria was also identified as a major challenge in IMCI implementation. Shortage of health care workers compared to very many children seeking for treatment was also identified.

It was reported by the district IMCI focal person that the City Council Medical Officer is supportive of IMCI training and this also could have led to better IMCI training coverage for the health care workers. The challenge is that the council budget is limited and therefore for training and there is no budget for IMCI follow-up and supportive supervision. The IMCI focal person reported that there is no budgetary allocation for IMCI and therefore IMCI follow up is combined with other Council Health Management Team (CHMT) routine follow-ups. The IMCI facilitators reported that they encounter challenges in following-up of trainees because the district councils do not have adequate budgetary allocation for follow-up and onsite mentorship. The City Medical Officer and the IMCI focal person reported that the Healthcare workers attitude change significantly to positive after these individuals received the two weeks IMCI training.

The district IMCI focal person also stated that it is a requirement to include at least one IMCI training every financial year in the Comprehensive Council Health Plan and budget for it to be approved. This requirement pushes the Council Health Management Team to include one training for 15 participants every financial year which has helped in training more health care workers on IMCI in Mwanza city.

The healthcare support system factors like shortage of supplies, lack of mentoring and supportive supervision were identified by the Mwanza city health administrators as the major factors that if addressed can lead to full implementation of IMCI by the health care workers.

### How can the IMCI training improved

The trained health care workers suggested the following points in improvement of IMCI training:

•Having refresher courses since there are a lot of changes in childhood illnesses and treatment; initial training should be longer than two weeks in workshop or have onsite/on job mentoring.

•The length of the training to be extended from the usual two weeks to one month to allow more comprehensive training and understanding. Some health care workers reported attending IMCI training for less than one week which they reported was inadequate since they were supposed to learn a lot of information within a very short time.

•The City Council Health Management Team needs to encourage the management of the health facility and all the healthcare workers on benefits of IMCI since some are resistant to change and they view IMCI approach negatively.

## Discussions

### Knowledge and attitudes of health care workers in the implementation of IMCI

The trained healthcare workers (51%) on IMCI approach is still below the WHO recommendation that at least 60% of health care workers seeing sick children in the health facilities are trained in IMCI. The coverage is below the recommended WHO coverage due to high cost of IMCI training [3]. However the Healthcare workers trained in Mwanza city is above the national coverage which is estimated to be 14% of trained healthcare workers as per June 2009.

Most of the health care workers (60%) trained received 2 weeks training in workshops, the first week being class work and the second week being practical at some selected practical sites. This was reported to be convenient by the health care workers but continuous onsite monitoring and supportive supervision after the 2 weeks IMCI training would be more efficient since the health care worker will learn more and better at the clinical site with support from mentors. The importance of onsite mentoring and supportive supervision is also supported by another study by Tanzania service provision assessment [12].

There are no opportunities for refresher courses or onsite mentoring possibly leading to knowledge decay of the learned IMCI approach. Also, a majority of the health care workers (69%) felt that the IMCI training they received was not adequate for them to follow it consistently. This may be because the training was short compared to the content learned therefore they were unable to have adequate time to practice skills during the training. It could also be due to of lack of onsite mentoring and refresher courses at the workplace to enhance the health care worker knowledge and skills.

### Factors that hinder health care workers in the implementation of IMCI

As summarized in Table 3, some of the factors includes the frequent changes and updates in management of childhood diseases without incorporating in IMCI guidelines was identified too as another hindering factor for health care workers in the implementation of IMCI guidelines in the healthcare facilities. The new malaria case management guidelines were identified to have conflicting information with IMCI guidelines. This possibly would discourage the health care workers not to follow IMCI guidelines consistently. A comprehensive review of the IMCI guidelines by the national IMCI team would ensure all current updates in management of childhood illnesses in the IMCI are incorporated in order to minimize conflicts with other existing case management guidelines.

**Table 3:** Thematic table indicating Systemic and programmatic factors that hinder IMCI implementation

**Theme**	**Responses**	**Frequency**
Factors related to the IMCI program.	• The new guideline for treatment of Malaria where the child with fever is not allowed to be given ALU (Artemisin Combined Therapy) until MRDT (Malaria Rapid Diagnostic test) test is done and is test positive. Therefore the IMCI need to be updated to reflect these changes. Also IMCI still recommends SP but the latest updates recommend ACT.	47 (30%)
	• Drug resistance to the recommended drugs according to IMCI guidelines (especially Septrin/Cotrimoxazole	6 (4%)
	• The guidelines have many classifications of illness and details therefore it becomes challenging when it is being referred to.	8 (5%)
Factors related to planning & allocation of resources.	• Shortage of essential drugs especially for treating pneumonia, diarrhea (ORS) and malaria.	27 (16%)
	• Shortage/ high turnover of health care workers compared to very many children seeking treatment.	32 (19%)
Factors related to Healthcare workers attitude.	• The IMCI approach is a very basic approach in the treatment of childhood illnesses.	3 (2%)
	• Lack of follow up (and if there not consistent) and adherence to IMCI guidelines.	17 (11%)
	• Referring to IMCI guidelines is boring (sometimes not user friendly i.e. it has too many details in one page).	5 (3%)
Factors related to parental/ care takers issues.	• Some mothers/care takers don’t believe their children have the samples taken to the medical lab to be tested for the right diagnosis and management (drugs) to be given	7 (4%)
	• Some mothers/care takers are not satisfied with the approach since most believe that the more drugs given to the child the better is the treatment/management. This is handled by giving health education to the mothers.	9 (6%)
TOTAL		161 (100%)

Shortage of supplies and essential drugs especially for treating pneumonia, diarrhea and malaria was also identified as a major challenge in IMCI implementation. Shortage of health care workers compared to the many children seeking treatment was noted to be a common factor hindering the implementation of IMCI in the health facilities. High turnover of health care workers was reported to be another factor which leads to shortage of staff. It is common for IMCI trained staff to leave their places of work for better opportunities in other districts or organizations. This can be solved by training more than 60% of the health care workers per WHO recommendations [3] so that even if there is high turnover there will still be trained staff to mentor untrained colleagues. Having good retention schemes can also make the health care workers not to leave the councils.

Lack of frequent follow up by IMCI focal person or CHMT is also an issue, as health care workers do not get adequate support in following of the IMCI guidelines at the health facilities. The Mwanza city council IMCI focal person reported that there is no budgetary allocation for IMCI and therefore IMCI follow up is combined with other CHMT routine follow-ups. Most health care workers (80%) were of the opinion that most in-charges and supervisors were not fully supportive in the implementation of IMCI and the reasons as to why health care workers felt their supervisors not fully supportive were lack of regular supportive supervision on IMCI and lack of mentoring on IMCI approach at the sites/health facilities. This might be discouraging health care workers in implementing IMCI guidelines and can be addressed by possibly educating them (supervisors) on the importance on IMCI, motivating them and clarifying their misconceptions regarding IMCI approach.

### Factors that would assist health care workers in the implementation of IMCI

As summarized in Table 4, several factors supporting the successful implementation of IMCI were identified by health care workers interviewed. Some of these factors are directly related to health care worker while others are related to healthcare system: Health care worker factor includes positive attitude in majority of the health care workers in the IMCI approach (Table 5). This positive attitude among the majority of the health care workers helps them to implement it irrespective of the associated challenges. Other studies [1,4] have also reported significant change in attitude after training of health care workers on IMCI approach. The factors related to health system were also identified as factors that might help in the implementation of IMCI. These include adequate supply of updated IMCI chart booklets and guidelines, onsite mentoring, conducting refresher courses for health care workers trained more than five years ago, consistent and supportive supervision from CHMT and district IMCI focal person and consistent supportive supervision.

**Table 4:** Thematic table indicating factors that could help in implementation of IMCI guidelines in the health facilities

**Theme**	**Responses**	**Frequency**
Factors that help in implementation of IMCI guidelines.	• IMCI guidelines available and booklets to be easily accessed in the offices/Clinical areas.	60 (30%)
	• Increasing the number of healthcare workers therefore reducing the workload, this will make the healthcare workers to follow the IMCI guidelines as recommended.	19 (10%)
	• On job Mentoring.	34 (17%)
	• Refresher courses.	20 (10%)
	• Supportive supervision	13 (6%)
	• Include IMCI in the continuing medical education series.	7 (4%)
	• IMCI training to be offered to all health care workers (staff workers and supervisors) and avail adequate working tools.	47 (23%)
TOTAL		200 (100%)

NB: The frequency total is more than the sample because all respondents gave more than one suggestion on what could help in IMCI implementation in the health facilities.

**Table 5:** Thematic table indicating Health care workers attitudes on IMCI approach

**Theme**	**Responses**	**Frequency**
**Attitudes and health care workers opinion on IMCI approach**.	• IMCI is very helpful since it can make health care workers to classify illness and treat the child even without laboratory investigations or sophisticated equipment’s.	39 (13%)
	• IMCI guidelines, booklets are very good if health care workers refer to when classifying illnesses then it can easily manage common childhood illnesses.	35 (12%)
	• Essential drugs (ACT for Malaria and Antibiotics) recommended in the guidelines are not always available so making the following of the guidelines difficult.	21 (8%)
	• There is need to have more IMCI refresher courses and on site mentoring.	32 (11%)
	• Need to have many guidelines and chart booklets for everyone so that they can refer to anytime they need.	60 (21%)
	• The health facilities in-charges and all the health care workers need to be educated on IMCI approach for all to have a common understanding.	15 (5%)
	• There is need to review and update the IMCI guidelines in line with the latest recommended drugs especially for malaria and pneumonia to be in line with changes in other guidelines produced by MOHSW (especially the one on malaria).	23 (8%)
	• There is need to increase the number of health care workers since workload is too much and therefore they tend to follow shortcut/not follow IMCI guidelines as it is recommended.	26 (9%)
	• IMCI guidelines need to be summarized more since it has a lot of explanations and details making it difficult to follow. It can be designed to be similar to the one of Syndromic Management of STI which seems to be more user-friendly.	20 (8%)
	• Cotrimoxazole out of experience does not work in treatment of pneumonia. It seems pneumonia infection have become more resistant to Cotrimoxazole.	5 (2%)
**TOTAL**		**276 (100**%**)**

Increase of workforce was also identified as a factor which will lead to effective implementation of IMCI guidelines by health care workers; this is because it will reduce workload and therefore give them more time to adhere to IMCI consistently in treatment of children. Shortage of health care workers has been identified in other studies as the major challenge in the implementation of IMCI in health centers and dispensaries [3,13,17-19].

These healthcare support system challenges are discouraging health care workers in the process of implementation of IMCI in their work place. Continuing professional development needs to be introduced in health facilities and IMCI needs to be one of the topics to be presented to health care workers frequently in order to help them understand and apply in managing childhood illness.

Adequate supply of essential drugs and supplies was also reported by health care workers as necessary to enable them to implement IMCI consistently. The common healthcare supplies which run out of stock at the health facilities frequently are essential drugs mainly amoxicillin, co-trimoxazole, ACT, Zinc Sulphate, chloramphenicol, gentamycin and x-pen. The other supply frequently not available includes oral rehydration salts (ORS), cups and buckets. Lack of these essential drugs and supplies recommended to be used as per IMCI guidelines may discourage the health care workers in the implementation of IMCI in their work place.

The need to strengthen capacity of health care workers to implement IMCI and support of the supply of essential drugs and supplies has also been noted by the government and other development partners. A 5 year USAID funded project in Lake Zone of Tanzania (Diagnosis and Management of Severe Febrile Illness) has been started with the goal of strengthening the IMCI implementation in Tanzania. Its key activities for the project include building the capacity of health care workers in order to diagnosis and treat child illness, and collaborating with the Tanzanian Food and Drug Administration (TFDA) to expand accredited drug dispensing outlets to ensure the supply of essential medicines [20].

The district IMCI focal person also stated that it is a requirement to include at least one IMCI training every financial year in the Comprehensive Council Health Plan and budget for it to be approved therefore ensuring IMCI training is one of the priority area.

The healthcare support system factors like shortage of supplies, lack of mentoring and supportive supervision have been also identified by other studies [21] as the major factors that if addressed can lead to full implementation of IMCI by the health care workers which is also concurred by this study.

### Limitations of the study

The following are the limitations for this study:

1. A major limitation of this study was its small sample size, over representation of health workers from Health Centers as opposed to dispensaries & sampling process (convenient) due to resource constrain, therefore making the study to be underpowered. A larger sample might have revealed more significant relationships. However, the sample size of 95 is large for a mixed methods study with resource constraints.

2. Not catering for design effects also affected the power of the study.

3. The study was done in Mwanza, and therefore the findings might not be generalized to other parts of the country since the settings of the study might not be entirely similar to other locations in Tanzania.

## Conclusions

The IMCI program is regarded positively by health care workers in Mwanza city and they are optimistic that full implementation of IMCI will occur if there is collaboration of supervisors, IMCI focal person, CHMT and other stakeholders interested in child health. However significant barriers impede a sustainable IMCI implementation.

These barriers identified by healthcare workers are: lack of supervision, insufficient number of trained health care workers, non-harmonization of malaria with IMCI guidelines, lack of motivation and retention of health care workers, shortage of essential drugs and supplies and poor attitude of some health care workers in the implementation of IMCI. Lack of supportive supervision and onsite mentoring makes the health care workers to continue with old ways of managing childhood conditions without integration as recommended in the IMCI approach.

There are no refresher courses for health care workers who have been trained on IMCI since the time IMCI was introduced (15 years ago) leading to knowledge decay. Finally there is shortage of essential drug and supplies in all the health facilities visited as recommended by IMCI case management guideline making full implementation of IMCI in health facilities difficult.

### Implications of the study

The overall benefit of IMCI is to contribute to the reduction of under 5 mortality rate by 2/3 which is in line with MDG 4. Understanding the reasons for non-adherence to the IMCI guidelines will help policy and decision makers to improve IMCI implementation in Tanzania and in other developing countries. The findings and recommendations will be made available to the Mwanza City Medical Officer. It will also be shared with other stakeholders who may include, interested development Partners, RMO, RCH, Health Centers and Dispensaries staff.

### Consent

All participants were provided with consent information sheet and forms to read and consent for participation by appending his/her signature in the provided consent form.

## Competing interests

The authors declare that they have no competing interests.

## Authors’ contributions

ABK: Main author of the study, involved in design, writing the proposal, supervised data collection, analysis and preparation of the manuscript. RM: Contributed to the designing of the proposal, data analysis and discussion. DMw: Involved in the developing the proposal & data collection tools, data analysis, interpretation of results and editing of the final manuscript. DMo: Involved in development of data collection tools, data analysis, and editing of the final manuscript. All the authors read and approved the final manuscript.

## Pre-publication history

The pre-publication history for this paper can be accessed here:

http://www.biomedcentral.com/1471-2458/14/277/prepub
